# How the Black Swan damages the harvest: Extreme weather events and the fragility of agriculture in development countries

**DOI:** 10.1371/journal.pone.0261839

**Published:** 2022-02-02

**Authors:** Nadine Marmai, Maria Franco Villoria, Marco Guerzoni

**Affiliations:** 1 DESPINA - Department of Economics and Statistics “Cognetti de Martiis”, University of Turin, Turin, Italy; 2 University of Modena and Reggio Emilia, Modena, Italy; 3 DEMS University of Milan, Bicocca, Italy; Universiti Teknologi Malaysia, MALAYSIA

## Abstract

Climate change constitutes a rising challenge to the agricultural base of developing countries. Most of the literature has focused on the impact of changes in the means of weather variables on mean changes in production and has found very little impact of weather upon agricultural production. Instead, we focus on the relationship between extreme events in weather and extreme losses in crop production. Indeed, extreme events are of the greatest interest for scholars and policy makers only when they carry extraordinary negative effects. We build on this idea and for the first time, we adopt a conditional dependence model for multivariate extreme values to understand the impact of extreme weather on agricultural production. Specifically, we look at the probability that an extreme event drastically reduces the harvest of any of the major crops. This analysis, which is run on data for six different crops and four different weather variables in a vast array of countries in Africa, Asia and Latin America, shows that extremes in weather and yield losses of major staples are associated events. We find a high heterogeneity across both countries and crops and we are able to predict per country and per crop the risk of a yield reduction above 90% when extreme events occur. As policy implication, we can thus assess which major crop in each country is less resilient to climate shocks.

## Introduction

This paper investigates the effect of temperature and precipitation extremes on major staple crops in different regions of Asia, Africa, and Latin America. We provide two key contributions to the literature. First, we adopt a conditional dependence model for multivariate extreme values developed by [1]. Although this modelling approach already has some applications in environmental or food chemicals studies, see e.g. [2, 3], there has been no previous attempt to use the model in the context of extremes in weather and crop production losses. Secondly, we provide evidence that these extreme events are associated and we are able to estimate their dependence structure.

The relation between climate and agriculture is a highly debated issue. By focusing on the agricultural sector of countries in Latin America, Africa and Asia, numerous studies discussed the impact on weather variables on food production [4–13]. For example, [4] found both positive and negative impacts of temperature and precipitation trends for different major crops at the global level. Specifically, trends in temperature affect mainly yield, whereas precipitation influences inter-annual changes in crop production. More recently, their estimates have been corroborated by [12].

This stream of research makes use of linear regression models and focuses on the mean effects of weather on average crop production [4, 14, 15]. The results of, e.g., [16] reveal that time constant country-fixed effects and time trends explain most of the variation in yields of different agricultural products. Regional characteristics such as soil quality or crop management and country-specific trends, e.g., technological progress in crop production or warming, are the most crucial factors, whereas annual mean changes in weather provide only a minor explanation of the overall variation. This approach suggests that the relation between weather and production is non-linear and difficult to model with linear regression analysis. [17] discuss the importance of non-linear responses to temperature in agricultural and non-agricultural production for both rich and poor countries: production peaks at an annual average temperature of 13 degrees Celsius and decreases substantially at higher temperatures. However, the average temperature is higher in poor countries, which leads to stronger effects of temperature on production in these countries. Projections suggest that further warming will reduce productivity and income in countries with high average temperatures.

The assessment of weather impacts on crop production includes not only the focus on changes in the mean values of weather variables but also on the probability, frequency, and severity of extreme events, which substantially influence yields [18–22]. Several empirical works, such as [23] in European countries, [24] in the United States, [25] in the UK, and [26] in France show a substantial impact of extreme weather events on the yields for major crops. Moreover, changes in the frequency of extreme weather events also determine the quality of the crop harvest [27]. [22] estimate the impact of heat stress and drought oat the global level over the period 1980–2010.

This evidence deserves a deeper analysis, since both the frequency and the magnitude of extreme weather events such as heat-waves are likely to rise due to warming climate conditions [25]. Looking at countries globally, [28] find extreme heat events to be unfavourable for major producing regions and lower income countries and, according to [25], extreme high and low temperature can seriously harm crops or even cause plant death, whereas intensive precipitation can lead to contamination of ground water and soil erosion. In addition to the direct effects of heat, drought, and flooding, extreme events indirectly affect crops through pests, changing soil processes and nutrient dynamics [29]. Similarly, [20] analyses the damages of extreme weather disasters on crop production and find that drought and extreme heat significantly harm national staple crop production worldwide. More recently, [30] make use of threshold effects of extreme weather events to estimate cereal yields in India.

Based on these studies, we surmise that the missing piece in the literature consists of relating the extreme events in weather with substantial losses in production. Indeed, extreme events are of the greatest interest for scholars and policy makers when they have extraordinary negative impacts [31]; otherwise the extreme events are irrelevant. Specifically, we test the hypothesis that the tails of weather variables and crops yields are associated. We also test that this association is heterogeneous across countries and staple crops. In the remainder of the paper, we model the conditional extreme dependence of extraordinary yields losses with four different weather variables for six different staple crops, separately for different regions in Asia, Africa and Latin America.

## Materials and methods

### Data

This study covers countries in Africa, Asia, and Latin America ranging from low to upper middle income countries [32] and includes annual data observations from 1961 to 2002. The staple crops of interest are wheat, rice, maize, soybeans, barley, and sorghum which constitute the six most commonly cultivated crops worldwide [6]. Country-level data on yields are available from the FAO website(http://faostat3.fao.org).

We make use of [4]’s precipitation and temperature data; the authors constructed weather data based both on [33]’s crop calendar to derive the growing season of each crop and on the agricultural maps of [34] to identify the growing regions of each crop. [4] extracted growing season- and region-specific weather data from the CRU TS 2.1 historical climate data set [35] and created national precipitation and temperature aggregates for each year. Different growing regions and different growing seasons among countries result in different precipitation and temperature data by crop and by country. Table 1 gives an overview of the variables.

**Table 1:** Overview of variables.

Variable	Description
*Yield*	Total production divided by area (hg/ha)
*Prec*	Total growing season precipitation in millimeters
*tMin*	Average minimum daily growing season temperature in degree Celsius
*tMax*	Average maximum daily growing season temperature in degree Celsius

The initial sample consists of 42 yearly observations for each country and each variable of analysis. We omit year-country pairs when data are missing for one of the variables. Because 42 observations constitute a small sample to conduct an analysis of extreme values, we pool countries into regions based on the UN Statistics Division composition of geographical regions (http://unstats.un.org/unsd/methods/m49/m49regin.htm). Table 2 summarizes the geographical areas, which consist of countries geographically related but still not identical in terms of economic and weather conditions. To account for this heterogeneity within each pool of countries, we standardize the production and weather data independently for each country, subtracting the country-specific mean and dividing by the country-specific standard deviation. This standardization ensures comparability and avoids missing extremes for some countries when pooling data.

**Table 2:** Geographical regions.

Region	Countries
South America	Argentina, Bolivia, Brazil, Chile, Colombia, Ecuador, Guyana, Paraguay, Peru, Suriname, Uruguay, Venezuela
Central America & Caribbean	Belize, Costa Rica, Cuba, Dominican Republic, El Salvador, Guatemala, Haiti, Honduras, Jamaica, Mexico, Nicaragua, Panama, Trinidad and Tobago
Western Africa	Benin, Burkina Faso, The Gambia, Ghana, Guinea, Ivory Coast, Liberia, Mali, Mauritania, Niger, Nigeria, Senegal, Sierra Leone, Togo
Eastern Africa	Burundi, Comoros, Djibouti, Eritrea, Ethiopia, Kenya, Madagascar, Malawi, Mozambique, Rwanda, Somalia, Sudan, Uganda, United Republic of Tanzania, Zambia, Zimbabwe
Middle & Southern Africa	Angola, Botswana, Cameroon, Central African Republic, Chad, Congo, Gabon, Lesotho, Namibia, South Africa, Swaziland
South & South-Eastern Asia & Melanesia	Bangladesh, Brunei, Bhutan, Cambodia, Indonesia, India, Fiji, Laos, Malaysia, Myanmar, New Caledonia, Papua New Guinea, Philippines, Solomon Is., Thailand, Vanuatu, Vietnam

**Fig 1:**
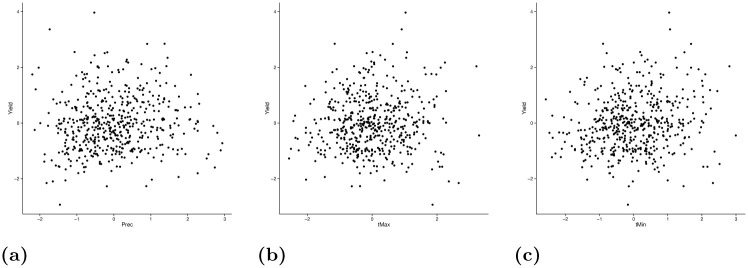
Annual standardized yield and precipitation (a), maximum temperature (b), and minimum temperature (c), using maize data in Eastern Africa (1961–2002).

Fig 1 shows scatter plots of the standardized yields *(Yield)*, precipitation *(Prec)*, maximum temperature *(tMax)* and minimum temperature (*tMin*), using maize data in Eastern Africa as a representative example of the data. Fig 1 shows that a regression analysis is not a convenient option because there are no grounds to assume a clear positive or clear negative dependence of the yield and any of the weather variables. Instead, we model the extremes, that is for instance, the set of observations in the bottom right corner of Fig 1a.

We define extreme events in precipitation, temperature, and yield as the values of the corresponding variable above (below) a threshold value, so that the observations subject to the analysis are located in the upper (lower) tail of the distribution [36]. We are mostly interested in the effect that extremely high or low temperatures and high or low precipitations have on the probability of observing severe yield losses.

### Methods

Let *X* = (*X*_1_, …, *X*_*d*_) be a vector variable with unknown distribution function *F*(*x*), and marginal distribution functions $F_{x_{i}}$, with *i* = 1, …, *d*. The idea is to model the joint tail of *F*(*x*) and, more specifically, the conditional distribution of *X*_−*i*_|*X*_*i*_ > *x* when *x* is large, where *X*_−*i*_ denotes the vector *X* excluding the *i*^*th*^ component. In this framework, we follow [1], who proposed a semi-parametric model ${\hat{F}}_{x_{i}}$ for the marginal distributions based on the generalized Pareto distribution (GPD),

$$\;{\hat{F}}_{x_{i}}=\{\begin{array}{cc} 1-\left\{1-{\tilde{F}}_{x_{i}}\left(u_{x_{i}}\right)\right\}{\left\{1+\xi_{i}\left(x-u_{x_{i}}\right)/\beta_{i}\right\}}_{+}^{-1/\xi_{i}} & x>u_{x_{i}}\\ {\tilde{F}}_{x_{i}}\left(x\right) & x\leq u_{x_{i}} \end{array}$$

where (*β*_*i*_,*ξ*_*i*_) are the scale and shape parameters of a GPD for the exceedances over the threshold $u_{x_{i}}$ and ${\tilde{F}}_{x_{i}}$ is the empirical distribution of *X*_*i*_.

Following [37], we use the estimated distributions ${\hat{F}}_{x_{i}}$ to transform *X* component-wise to follow Laplace marginal distributions:

$$\begin{array}{c} Y_{i}=\{\begin{array}{cc} \text{log}\{2F_{X_{i}}\left(X_{i}\right)\} & \text{for}\;X_{i}<F_{X_{i}}^{-1}\left(0.5\right)\\ -2\text{log}\{2[1-F_{X_{i}}\left(X_{i}\right)]\} & \text{for}\;X_{i}\geq F_{X_{i}}^{-1}\left(0.5\right). \end{array} \end{array}$$ (1)

The aim is to model the distribution of *Y*_−*i*_|*Y*_*i*_ = *y* for *y* large. For that purpose, univariate extreme value theory is extended to a multivariate context. Assume that there exist normalizing functions *a*_|*i*_(*x*), *b*_|*i*_(*x*): $R\to R^{d-1}$ / ∀ fixed $z\in R^{d-1}$ and any sequence of *y*_*i*_ values such that *y*_*i*_ → ∞ (i.e., high enough):

$$\begin{array}{c} lim_{y_{i}\to \infty }\left[Y_{-i}\leq a_{|i}\left(y_{i}\right)+b_{|i}\left(y_{i}\right)z_{|i}|Y_{i}=y_{i}\right]=G_{|i}\left(z_{|i}\right). \end{array}$$ (2)

Denote by *G*_*i*_ the *i*^*th*^ marginal distribution of *G*_|*i*_, a non-degenerate distribution function with *lim*_*z*→∞_{*G*_*i*_(*z*)} = 1 ∀*i*. The method assumes that the limiting distribution holds ∀ $y_{i}>u_{Y_{i}}$ for a suitable high threshold $u_{Y_{i}}$. When *Y*_*i*_ = *y*_*i*_, with $y_{i}>u_{Y_{i}}$, the (standardized) random variable *Z*_|*i*_ is defined as:

$$\begin{array}{c} Z_{|i}=\frac{Y_{-i}-a_{|i}\left(y_{i}\right)}{b_{|i}\left(y_{i}\right)} \end{array}$$ (3)

and the limiting distribution of *Z*_|*i*_:

$$\begin{array}{c} lim_{y_{i}\to \infty }P\left(Z_{|i}\leq z_{|i}|Y_{i}=y_{i}\right)=G_{|i}\left(z_{|i}\right). \end{array}$$ (4)

Under this assumption, conditionally on $Y_{i}>u_{Y_{i}}$, as $u_{Y_{i}}\to \infty$, the variables $Y_{i}-u_{Y_{i}}\left(>0\right)$ and *Z*_|*i*_ are independent in the limit and their limiting marginal distributions are exponential and *G*_|*i*_(*z*_|*i*_), respectively [37].

The extremal dependence behaviour is characterized by *a*_|*i*_(*y*), *b*_|*i*_(*y*) and *G*_|*i*_; hence estimates of the three are needed to derive the conditional distribution. To do so, [1] propose a semi-parametric model. The parametric part involves estimating *a*_|*i*_(*y*) and *b*_|*i*_(*y*) using the regression model:

$$\begin{array}{c} Y_{-i}=a_{|i}\left(y\right)+b_{|i}\left(y\right)Z_{|i}=a_{|i}y+y^{b_{|i}}Z_{|i}. \end{array}$$ (5)

Specifically, *a*_|*i*_(*y*) and *b*_|*i*_(*y*) are expressed in terms of *y* as *a*_|*i*_(*y*) = *a*_|*i*_ *y* and $b_{|i}\left(y\right)=y^{b_{|i}}$, with the restrictions (*a*_|*i*_, *b*_|*i*_) ∈ [−1, 1]^*d*−1^ × (−∞, 1)^*d*−1^. Further joint constraints on the parameters have been imposed by [37] to avoid problems of inconsistent inferences with respect to the marginal distributions and parameter identification. Positive and negative dependence are defined by *a*_*j*|*i*_, the component of *a*_|*i*_ linked to *Y*_*j*_ and large *Y*_*i*_, being 0 < *a*_*j*|*i*_ ≤ 1 and −1 ≤ *a*_*j*|*i*_ < 0 respectively [37]. Assuming that (*a*_|*i*_, *b*_|*i*_) are known, *G*_|*i*_ can be estimated non-parametrically using the empirical (or kernel smoothed) distribution of replicates of the random variable ${\hat{Z}}_{|i}$:

$${\hat{Z}}_{|i}=\frac{Y_{-i}-{\hat{a}}_{|i}\left(y_{i}\right)}{{\hat{b}}_{|i}\left(y_{i}\right)}$$

for $Y_{i}=y_{i}>u_{Y_{i}}$. Pseudo-samples can then be generated using the fitted model to estimate the conditional probability of interest. Confidence intervals can be obtained using bootstrap methods, see [1] for computational details.

## Results

### Empirical analysis

In the case of *Prec*, we consider both extreme high and low precipitation values. Because [1] model the upper tail of the distribution, for extreme low precipitation, we consider the reflection of the variable *Prec*, defined as *PrecRefl*. The same procedure applies to *Yield* because we are interested in yield losses and we therefore consider the reflection *YieldRefl*. For each crop, we define four two-dimensional vectors *X* = (*X*_1_, *X*_2_) with unknown distribution function *F*(*x*), where *X*_1_ is always *YieldRefl* of the specific crop and *X*_2_ is one of the four weather variables. We thus model the extreme values in a bivariate context and run a separate analysis for each pair of crop and weather variables. To illustrate the procedure of fitting the dependence model, we report results of the two variables *YieldRefl* and *Prec* using maize data of Eastern Africa from 1961 to 2002. Results for the set of specifications with different marginal weather variables and for different crops or regions are available on request. The analysis was conducted using the R packages texmex [38], evd [39], ggplot2 [40] and rworldmap [41].

We first fit a generalized Pareto distribution separately for each variable and then transform *X* component-wise following Eq (1) to obtain identical Laplace marginal distributions. The fitting of the GPD requires an appropriate selection of the threshold above which the GPD model is valid. For this reason, linearity of the mean residual life plots has to be ensured, which is already the case for very low thresholds of *Prec* and *YieldRefl* for maize data in Eastern Africa. However, probability, quantile and return level plots suggest that the estimated distribution function is a reasonable estimate of the theoretical function only above the threshold of the 80^*th*^ percentile [36], which we then choose as the threshold for *Prec* and *YieldRefl*.

**Fig 2:**
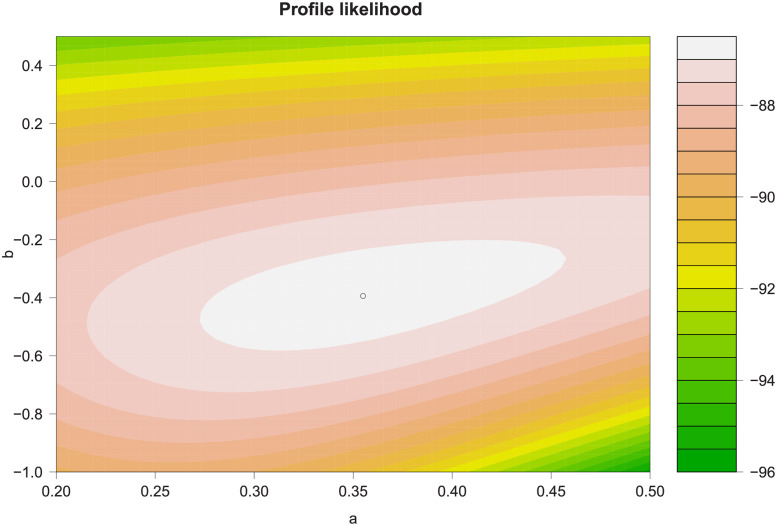
Constrained dependence parameter estimates ${\hat{a}}_{j|i}$ (a) and ${\hat{b}}_{j|i}$ (b) of the conditional distribution of (*YieldRefl*|*Prec*) conditional on *Prec* > *qu*_*Prec*_, with *qu*_*Prec*_ being the 90^*th*^ quantile of the conditioning variable *Prec*, correspond to the maximum of the profile likelihood surface using maize data of Eastern Africa.

Based on these marginal variables with identical Laplace distributions, we describe the dependence structure of the variables. We explore the behaviour of the variable *YieldRefl* conditional on extreme values of the variable *Prec*. We choose the threshold $u_{Y_{i}}$ over which the limiting distribution holds by examining the threshold stability of the estimated parameters *a*_|*i*_ and *b*_|*i*_ of the dependence model (5) using the 50^*th*^ to 90^*th*^ quantiles of the conditioning variable *Prec* as potential thresholds. The 90^*th*^ quantile was found to be adequate, leading to parameter estimates ${\hat{a}}_{j|i}=0.355$ and ${\hat{b}}_{j|i}=-0.394$. Imposing the ordering constraints to the values of the parameters as explained in Section Methods, parameter estimates can be sensitive to the initial value of the optimization procedure for the estimation [42]. However, constrained dependence parameter estimates are located in the maximum of the profile likelihood surface, as shown in Fig 2. With ${\hat{a}}_{j|i}=0.355$, the variables *YieldRefl* and *Prec* exhibit positive extremal dependence [37]. Using maize data from Eastern Africa, we see that the form of dependence between *YieldRefl* and the weather variables varies. In contrast to *YieldRefl* and *Prec*, the pair *YieldRefl* and *PrecRefl* shows an extremal negative dependence with an ${\hat{a}}_{j|i}=-0.6265$. The results of the other regions and for the other crops show that the dependence structure does not only change for different conditioning variables but also for different crops and regions. The uncertainty of the parameter estimates was evaluated by means of bootstrapping, see [1] for details.

Finally, plotting *Z*_|*i*_ against *Y*_*i*_ for values of *Y*_*i*_ over the threshold chosen to fit the model, we conclude that the standardized variable and the conditioning variable are independent. Moreover, the fitted quantiles of the conditional distribution and the observations on the original scale match indicating that the model fits well.

### Extrapolation

The semiparametric model fitted in the previous section is used to simulate from the joint distribution of (*YieldRefl*, *Prec*) conditional on *Prec* > *qu*_*Prec*_, where *qu*_*Prec*_ is the 91^*st*^ to 99,99^*th*^ quantile of the conditioning variable *Prec*. We simulate 1000 observations, which are then used to calculate the conditional probabilities *P*(*YieldRefl* > *qu*_*YieldRefl*_|*Prec* > *qu*_*Prec*_), where *qu*_*YieldRefl*_ is always set as the 90^*th*^ quantile of the variable *YieldRefl*. Thus, we are interested in the change of the probability of high losses in basic food production given rising extremes in precipitation. The uncertainty of each point estimate is assessed by creating 95% confidence intervals of the conditional probabilities based on 100 bootstrap samples.

**Fig 3:**
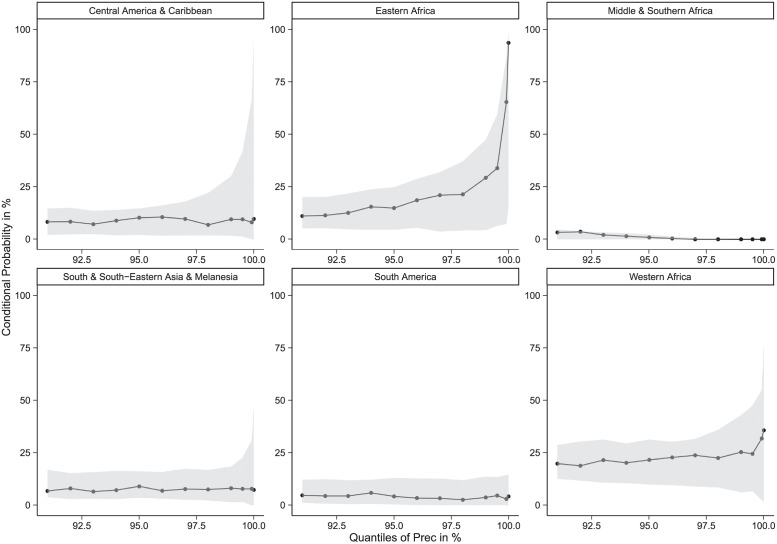
Point estimates and 95% confidence intervals of the conditional probability *P*(*YieldRefl* > *qu*_*YieldRefl*_|*Prec* > *qu*_*Prec*_), where *qu*_*YieldRefl*_ is always set as the 90^*th*^ quantile of the variable *YieldRefl* and *qu*_*Prec*_ is the 91^*st*^ to 99,99^*th*^ quantile of the conditioning variable *Prec*. Estimation is done using maize data from 1961 to 2002. In Eastern Africa conditional probabilities sharply increase with widening confidence intervals for high thresholds of the conditioning variable. In Middle & Southern Africa the conditional probabilities or the lower confidence interval bounds are zero indicating no evidence of an association between extremes in high precipitation and high yield losses.

Fig 3 contains conditional probabilities and the corresponding confidence intervals of high losses in maize production given a range of thresholds, i.e. from 91^*st*^ to 99.99^*th*^ quantiles, for high precipitation. The plots in Fig 3 cover all considered regions in Asia, Africa and Latin America. In Middle & Southern Africa, the conditional probabilities of lower confidence interval bounds are equal to zero, indicating no evidence of an association between extremes in high precipitation and high yield losses. The probability of high losses in maize production given increasing extremes in high precipitation increases sharply up to 94% in Eastern Africa but with widening confidence intervals. Eastern Africa aside, the probabilities do not change significantly with increasing thresholds for high precipitation among the different regions and are mostly below 25%. For the opposite case of extremes in low precipitation, Fig 6 in S1 Annex plots the conditional probabilities for all regions. South America is affected more than the other regions because losses in its maize production are more likely to occur given extremely low rainfall. Point estimates up to 74% show higher uncertainties, although the confidence intervals do not include zero. In Central America & Caribbean, the lower confidence interval bounds of the conditional probabilities are equal to zero. The conditional probabilities for Eastern Africa are approximately 25% and are less than 25% for the other regions. Summing up, events of extremely high precipitation affect maize production the most in Eastern Africa and the least in Middle and South Africa, while drought has severe consequences on Maize production in South America and limited ones in In Central America & Caribbean.

In the case of extremes in minimum temperature, see Fig 7 in S1 Annex, the lower confidence interval bounds are equal to zero in South America, Western Africa and South & South-Eastern Asia & Melanesia. The other regions do not show remarkable differences. In Central America & Caribbean and Eastern Africa, the conditional probabilities slightly increase with higher extremes in minimum temperature. However, the point estimates are around or below 25%. The picture looks similar for the maximum temperature as the conditioning variable, which is shown in Fig 8 in S1 Annex. The conditional probabilities rarely exceed 25%, and the lower confidence interval bounds are equal to zero in Western Africa and South & South-Eastern Asia & Melanesia. The conditional probability plots for barley, rice, sorghum, soy and wheat also reveal a mixed picture and are shown in the supporting material to this paper [43, pp. 20–35]. Production losses due to weather extremes are not equally likely among the regions. Depending on the crop, the weather extremes and the region, the occurrence of severe production losses is more or less likely. The results emphasize the complexity of interaction factors.

**Fig 4:**
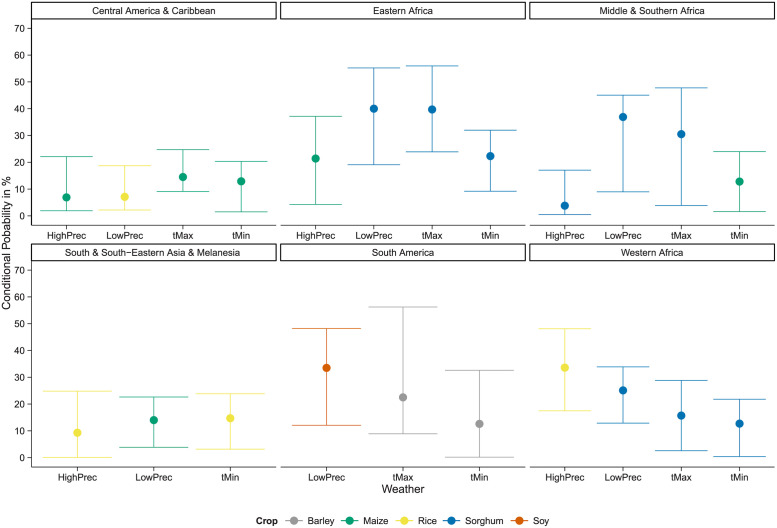
Highest point estimates of the conditional probabilities, i.e. the probabilities of yield losses above the 90^*th*^ quantile given minimum temperature, maximum temperature or high precipitation extremes above the 98^*th*^ quantile or low precipitation extremes below the 2^nd^ quantile. If the 95% confidence interval includes zero, the conditional probabilities are not shown.

Fig 4 visually summarizes this heterogeneity and displays the highest point estimates and the 95% confidence intervals of the probability of observing a high loss in yield, i.e., the probability to observe a reduction of Yield below the 10%, conditional to the occurrence of a weather extreme in the 98^*th*^ quantile.

Fig 4 shows that each of the considered regions is likely to have severe losses in its production of staple food due to extreme weather events. However, the effect of extreme weather varies among regions depending on the crop and the type of weather extreme. Sorghum, which is the main staple in Africa, has the highest risk of severe yield losses for different weather extremes in the three African regions. Extreme high maximum temperature and low precipitation are the main weather conditions leading to extraordinary yield losses, which is in line with the distribution of sorghum in arid regions in Africa or in regions where precipitation is erratic and characterized by short periods of high precipitation [44]. In Eastern and Western Africa extreme high minimum temperature lead to severe reduction in sorghum yield. High losses in maize production are due to extreme high minimum temperature in Middle & Southern Africa and are due to extreme high precipitation in Eastern Africa. Noteworthy because of the importance of rice, which is a main staple in Western Africa [45], high losses in rice production are likely to occur with extreme events in high precipitation.

Central America & Caribbean exhibits the highest conditional probabilities due to weather extremes for rice and maize, whereas South America shows it for barley and soybean which are among the most important staple crops in the regions. Weather patterns are diverse in Latin America. Whereas South America experiences rising temperature and changing precipitation patterns leading to mixed effects on agriculture, an increase in temperature severely damages agricultural output in Central America. Moreover, even though Central America is marked by a decline in precipitation, floods remain among the most frequent extreme weather events [46]. The estimated conditional probabilities suggest that in Central America & Caribbean maize production reacts mainly to extremes in high precipitation and temperature and rice production reacts to extreme low precipitation. In South America, barley and soybean production losses are likely to increase with extremes in temperature and low precipitation respectively.

In Asia a main staple crop is rice where 90% of rice production and consumption is concentrated [45]. The dependency on rice is reflected by the fact that we obtain the highest conditional probabilities for rice in South & South-Eastern Asia & Melanesia. Losses in rice production are likely due to extreme high minimum temperature. The finding is in line with [45] who state that higher minimum temperatures become increasingly a major cause of yield losses of rice in Asia. On the other side, the occurrence of very low rice yields is likely due to high precipitation. Only in South Asia yield losses of rice due to floods are about 4 million t per year [45]. Interestingly, the probability of production losses given extreme low precipitation is the highest for maize in the tropical region of South & South-Eastern Asia & Melanesia. The finding is plausible as maize production is predominantly rain-fed in South and South-Eastern Asia [47].

The point estimates of conditional probabilities shown in Fig 4 are at maximum 40%, which is the case for sorghum in Eastern Africa given extreme low precipitation. Overall, the highest conditional probabilities given different weather extremes are in Eastern and Middle & Southern Africa as well as in South America. In contrast, the conditional probabilities are less than 30% for all types of weather extremes in Central America & Caribbean and South & South-Eastern Asia & Melanesia.

Summing up the results, we find that, first, maize and sorghum have the highest conditional probabilities of extreme high losses in crop production given the occurrence of extreme weather conditions. Second, extreme low precipitation and extreme high maximum temperature are the defining weather extremes, except for Latin America and Western Africa. In Latin America, the probabilities do not change significantly among different conditioning variables, and in Western Africa, the highest conditional probability is due to extreme high precipitation in the case of rice. Third, losses in staple crop production given extreme weather events are more likely in the African regions and South America.

**Fig 5:**
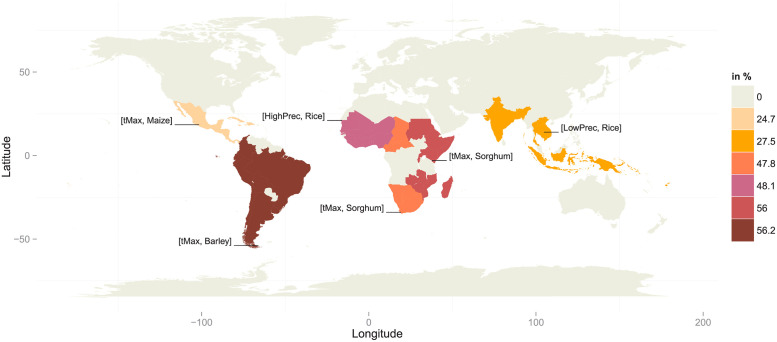
Worst-case scenario: The maximum upper bound of the 95% confidence interval of the conditional probability estimates, i.e. the probability estimates of yield losses above the 90^*th*^ quantile given minimum temperature, maximum temperature or high precipitation extremes above the 98^*th*^ quantile or low precipitation extremes below the 2^nd^ quantile.

Finally, Fig 5 shows the worst-case scenario by displaying the maximum upper bound of the 95% confidence interval of the conditional probability estimates, i.e., the probability estimates of yield losses above the 90^*th*^ quantile given the minimum temperature, maximum temperature or high precipitation extremes above the 98^*th*^ quantile or low precipitation extremes below the 2^nd^ quantile. The maximum upper bound of the 95% confidence interval is chosen from all of the weather variables and crops in a region. Each region has a different worst-case scenario, i.e., a different crop and weather variable for which we obtain the maximum upper confidence interval bound. In Central America & Caribbean, South America, Eastern Africa and Middle & Southern Africa, extreme high temperature is the condition that leads to the highest production losses. Whereas in the African regions, the associated crop is sorghum, the associated crop is maize in Central America & Caribbean and barley in South America. The worst-case scenario for Western Africa constitutes high losses in rice yields due to extremes in high precipitation. In the Asian region, extremes in low precipitation is the defining weather variable in the worst-case scenario and rice is the affected crop. The worst-case scenarios occur with different probability in the different regions. The probability ranges from around 25% in Central America & Caribbean and South & South-Eastern Asia & Melanesia to 56% in Eastern Africa and South America, whereas probabilities of approximately 50% are found in Western and Middle & Southern Africa.

This worst case scenario can be employed as a fragility index which can be used to assess strengths and weakness of different areas conditional to the type of crops they mostly harvest. It can thus became a valuable instrument for policy design. As a matter of fact, the same index can be applied to different areas other than agriculture, but still linked with climate change, such as desertification, diffusion of deceases, and poverty rate.

## Discussion and conclusion

The influential paper on African countries by [16] reports an expected 7% reduction of aggregate agriculture productivity with a probability of 95% probability due to change in the mean of weather variables in Sub-Saharan Africa with an R-squared between 0.5 and 0.7 according to the type of staple. Their work, as any other standard approach for assessing the impact of weather on crops has largely focused on the mean values. In line with a recent stream of research on extreme weather events, we do not focus on the mean but on the tails of the distributions. We find systematic evidence that extreme losses in production of major food staples are likely to occur in times of extreme conditions in weather and the probability depends on the type of weather extreme, the crop, and the region. The alleged increase in the number of extreme events should be taken seriously because the potential damage can be extraordinary. As a comparison with [16], independently on the mean, seasonal extreme events of drought can reduce Sorghum production in Sub-Saharan Africa by 40%, that is 7 times higher than the projected reduction of 7%. We are able to provide this measure of risk for each region, that is specific to both the crop and the extreme weather event. This outcome can also be used as a index for the resilience of a region and to help policy makers to intervene and set priorities. In the short run, a country cannot change the risk of incurring high losses due to weather events. However, the resilience of a country depends on many factors, such as the adoption of advanced irrigation technology, the diffusion of fertilizer, and the introduction of new and resistant crops, which can be highly influenced by the policy makers. This work is highly complementary with the ones focusing on the mean impact of the present trend of weather variables. While these works [6, 16, 24, 48, 49] can provide an assessment of the future long term development on average, we highlight that this path will be punctuated by events with a much higher disrupting effect. Thus, long term policy for climate change intervention should be accompanied by immediate action for counteracting the impact of very likely extreme events.

This work leaves some questions unanswered due to data availability. Specifically, the same type of weather extremes can lead to different crop responses based on the time of the year and the crop growth stage [26]. The use of growing-season national aggregates of weather to account for weather extremes instead of using precise weather data is critical because growing-season aggregates do not to capture inter-annual ups and downs and extremes within a growing period. The drawback occurs because extreme weather conditions are particularly harmful in certain stages of plant growth [50]. In addition, studies that focus on the occurrence of extreme weather events have to be accompanied by studies that also include the severity of extremes, such as the length of heatwaves or floods. The main constraint for a large fraction of countries worldwide is the lack of access to long-term weather data with high time and spatial resolution [51], which are now available for case studies limited in time and space. We try to mitigate this problem by looking at aggregates of weather in the growing season and for different growing regions and we analyse the occurrence of periods of extreme hot, dry or wet conditions from a long-term perspective. The value of this paper is to provide an indication of the probability of extreme losses in basic food production due to extreme weather events in a specific region under historic climate conditions. This analysis provides a perspective that is complementary to more detailed localized studies focusing on one particular region with higher time and spatial resolution data.

To conclude, studying weather changes and the impacts on agricultural production remains a challenging task. To evaluate crop production responses the uncertainty of weather changes needs to be further addressed. The uncertainty in the evaluation of current and future impacts of weather on agricultural production stems from uncertainties that arise in the estimation of crop responses to changes in average growing season temperature and precipitation [48]. We believe that the uncertainties also come from the extreme events that are by nature difficult to model. Given that future climate is likely to be prone to a higher frequency of extreme weather events, our results contribute to this discussion by providing the probabilities of severe staple crop production losses conditioned on extremes in temperature and precipitation.

## Supporting information

S1 Annex (ZIP)
