# Supplementary material for: How the Black Swan damages the harvest: Extreme weather events and the fragility of agriculture in development countries
Source: PLoS One. 2022 Feb 2;17(2):e0261839. doi: 10.1371/journal.pone.0261839 (PMC8809593; doi:10.1371/journal.pone.0261839)
Supplement: S1 Annex — (ZIP) [file pone.0261839.s001.zip › S1_Annex.pdf]

Annex

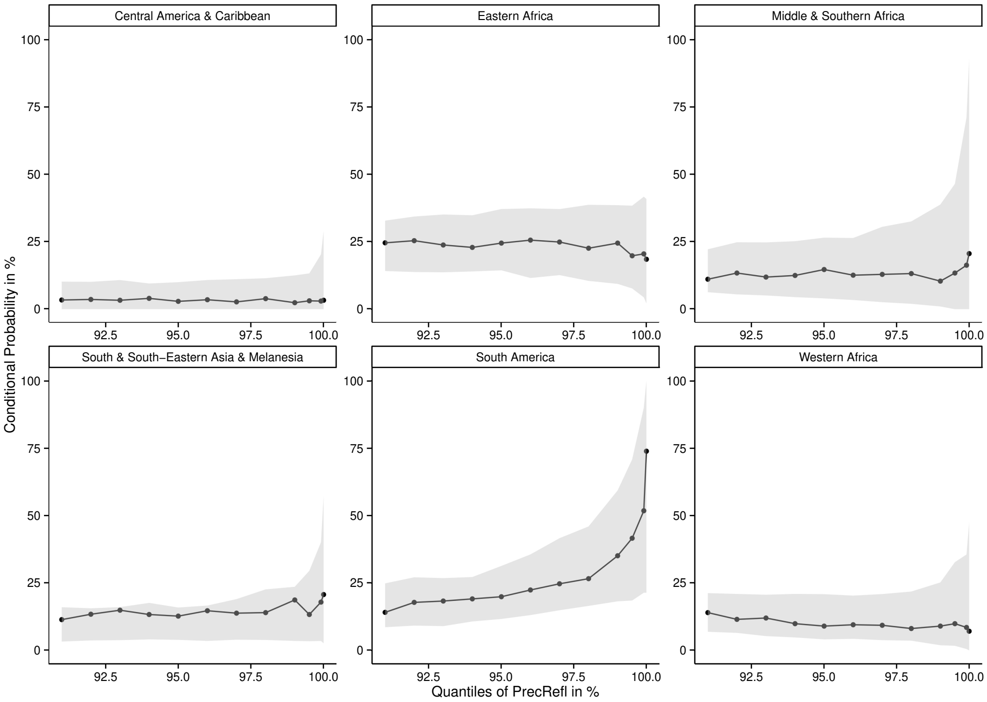

Fig 6. *Point estimates and 95 % confidence intervals of the conditional probability* $P(YieldRefl > qu_{YieldRefl}|PrecRefl > qu_{PrecRefl})$*, where* $qu_{YieldRefl}$ *is always set as the* 90th *quantile of the variable YieldRefl and* $qu_{PrecRefl}$ *is the* 91st *to* $99{,}99^{th}$ *quantile of the conditioning variable PrecRefl. Estimation is done using maize data from 1961 to 2002. In Central America & Caribbean the lower confidence interval bounds of conditional probabilities are equal to zero.*

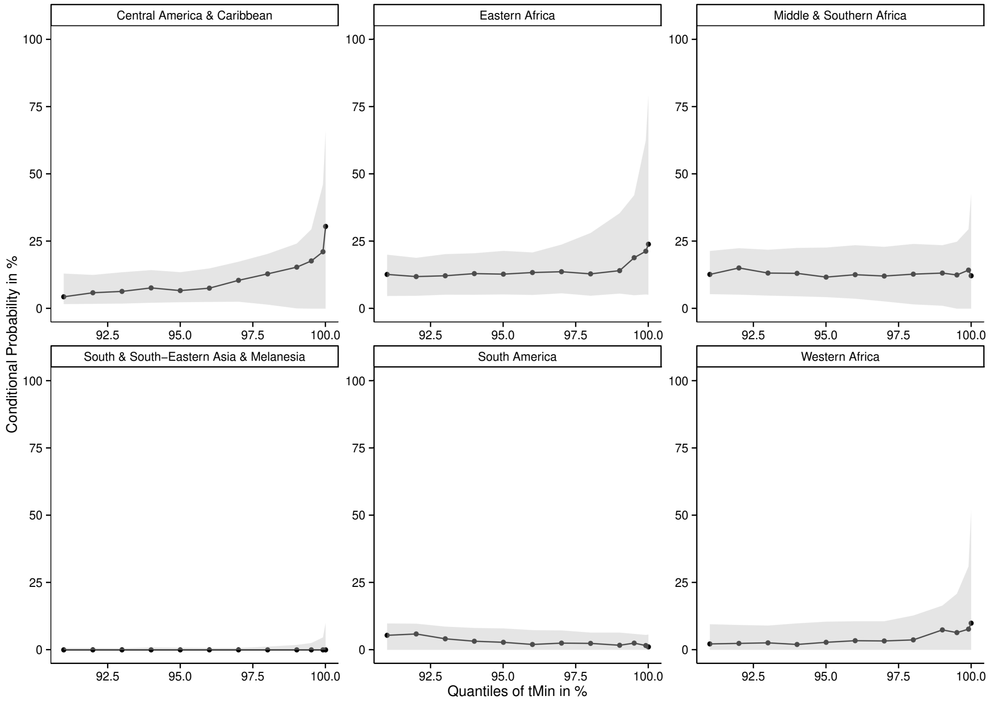

Fig 7. *Point estimates and 95 % confidence intervals of the conditional probability* $P(YieldRefl > qu_{YieldRefl}|tMin > qu_{tMin})$*, where* $qu_{YieldRefl}$ *is always set as the* 90th *quantile of the variable YieldRefl and* qu_{tMin} *is the* 91st *to* $99{,}99^{th}$ *quantile of the conditioning variable tMin. Estimation is done using maize data from 1961 to 2002. In South America, Western Africa and South, South-Eastern, and Eastern Asia & Melanesia the conditional probabilities or the lower confidence interval bounds are zero indicating no evidence of an association between extremes in minimum temperature and high yield losses.*

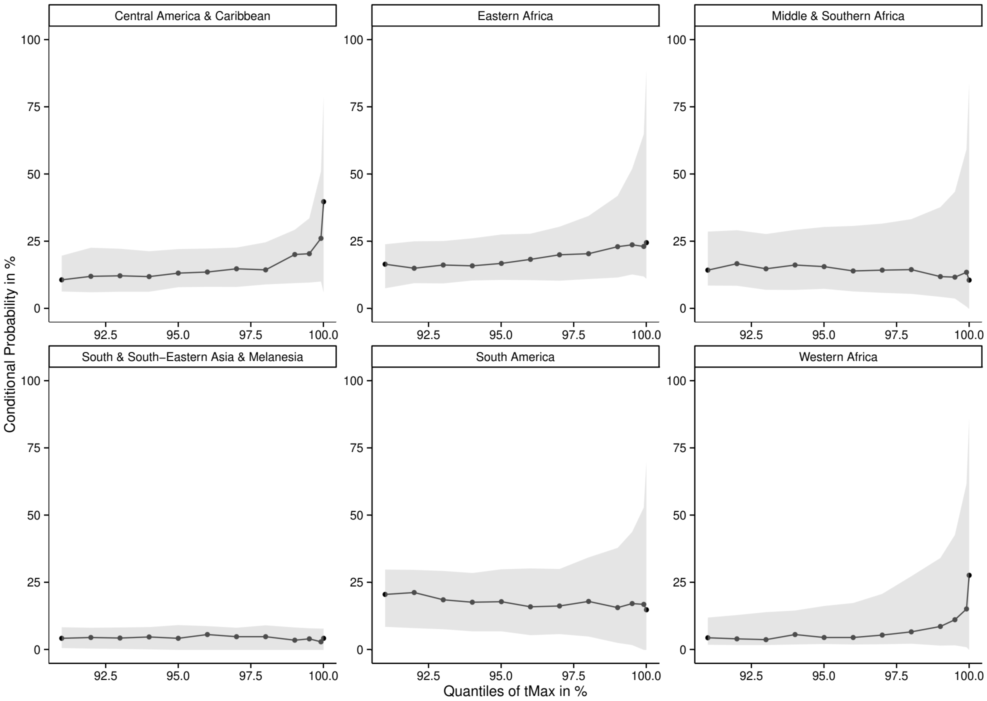

Fig 8. *Point estimates and 95 % confidence intervals of conditional probability* $P(YieldRefl > qu_{YieldRefl_i} = 90\% | tMax > qu_{tMax_i})$ *per region i. Estimation done using maize data from 1961 to 2002. In South & South-Eastern Asia & Melanesia the lower confidence interval bounds of conditional probabilities are equal to zero.*
